# Entrainment of neural oscillations during language processing in Early-Stage schizophrenia

**DOI:** 10.1016/j.nicl.2024.103695

**Published:** 2024-10-28

**Authors:** Tineke Grent-’t-Jong, Pradeep Dheerendra, Paolo Fusar-Poli, Joachim Gross, Andrew I. Gumley, Rajeev Krishnadas, Lars F. Muckli, Peter J. Uhlhaas

**Affiliations:** a Department of Child and Adolescent Psychiatry, Charité Universitätsmedizin, Berlin, Germany; b School of Psychology and Neuroscience, University of Glasgow, UK; c Department of Brain and Behavioral Sciences, University of Pavia, Pavia, Italy; d Early Psychosis: Interventions and Clinical-detection (EPIC) Lab, Department of Psychosis Studies, King’s College London, UK; e Department of Brain and Behavioral Sciences, University of Pavia, Italy; f Outreach and Support in South-London (OASIS) service, South London and Maudlsey (SLaM) NHS Foundation Trust, UK; g Department of Psychiatry and Psychotherapy, University Hospital, Ludwig-Maximilian-University (LMU), Munich, Germany; h Institute for Biomagnetism and Biosignalanalysis, University of Muenster, Muenster, Germany; i Institute of Health and Wellbeing, Univ. of Glasgow, UK; j Dept. of Psychiatry, Univ. of Cambdrige, Cambridge, UK

**Keywords:** Schizophrenia, Language, Entrainment, Neural oscillations, Clinical high-risk, MEG

## Abstract

**Background:**

Impairments in language processing in schizophrenia (ScZ) are a central aspect of the disorder but the underlying pathophysiology mechanisms are unclear. In the current study, we tested the hypothesis that neural oscillations are impaired during speech tracking in early-stage ScZ and in participants at clinical high-risk for psychosis (CHR-P).

**Method:**

Magnetoencephalography (MEG) was used in combination with source reconstructed time-series to examine delta and theta-band entrainment during continuous speech. Participants were presented with a 5-minute audio recording during which they either attened to the story or word level. MEG-data were obtained from n = 22 CHR-P participants, n = 23 early-stage ScZ-patients, and n = 44 healthy controls (HC). Data were analysed with a Mutual Information (MI) approach to compute statistical dependence between the MEG and auditory signal, thus estimating individual speech-tracking ability. MEG-activity was reconstructed in a language network (bilateral inferior frontal cortex [F3T; Broca’s], superior temporal areas [STS3, STS4; Wernicke’s areas], and primary auditory cortex [bilateral HES; Heschl’s gyrus]). MEG-data were correlated with clinical symptoms.

**Results:**

Theta-band entrainment in left Heschl’s gyrus, averaged across groups, was significantly lower in the STORY compared to WORD condition (p = 0.022), and averaged over conditions, significantly lower in CHR-Ps (p = 0.045), but intact in early ScZ patients (p = 0.303), compared to controls. Correlation analyses between MEG data and symptom indicated that lower theta-band tracking in CHR-Ps was linked to the severity of perceptual abnormalities (p = 0.018).

**Conclusion:**

Our results show that CHR-P participants involve impairments in theta-band entrainment during speech tracking in left primary auditory cortex while higher-order speech processing areas were intact. Moreover, the severity of aberrant perceptual experiences in CHR-P participants correlated with deficits in theta-band entrainment. Together, these findings highlight the possibility that neural oscillations during language processing could reveal fundamental abnormalities in speech processing which may constitute candidate biomarkers for early detection and diagnosis of ScZ.

## Highlights

• Clinical high risk for psychosis (CHR-P) participants were impaired in theta-band speech-tracking ability in primary auditory cortex.
• In schizophrenia patients, speech-tracking ability was intact.
• The severity of aberrant perceptual experiences in CHR-P participants correlated with their perceptual aberrations.
• These findings highlight the possibility that neural oscillations could reveal abnormalities in speech processing in psychosis.

## 1 Introduction

Despite progress in the identification of the neurobiological signatures of ScZ, the pathophysiological mechanisms that underlie major symptoms and cognitive impairments remain unclear (Tandon, 2024). Moreover, robust biomarkers that could facilitate diagnosis and prognosis as well as early detection are currently not available (Kraguljac, 2021). Recent advances in the computational and neurophysiological mechanisms of language processing, however, have the potential to address these central issues (Brown and Kuperberg, 2015, Chang, 2022).

Language anomalies are an essential aspect of ScZ and have been considered a core feature of the disorder since the early formulation of the clinical concept (Crow, 2008, Bleuler, 1950). Auditory hallucinations (AH) are present in 60–80 % of ScZ-patients that frequently involve the perception of speech (Lim, 2016). Moreover, formal thought disorder (FTD) refers to abnormalities in the content and form of speech and comprises positive, negative, and disorganized dimensions (Yalincetin, 2017). FTD is present in participants at clinical high-risk for psychosis (CHR-P) and FTD-severity predicts transition to psychosis (Perkins, 2015).

In regard to language perception, deficits in the processing of phonemes (Cienfuegos, 1999), syllables (Bruder, 2001) and prosody (Kantrowitz, 2015) have been demonstrated that correlate with functional impairments in ScZ-patients (Hooker and Park, 2002, Bonfils, 2019). However, not all studies have observed low-level speech perception deficits (Haigh, 2019) and it is unclear how impairments in the processing of syllables and phonemes, for example, relate to language comprehension (Brown and Kuperberg, 2015).

Recent work in cognitive and computational neuroscience has provided novel candidate mechanisms that could provide a unifying framework for altered language processes in ScZ (Brown and Kuperberg, 2015, Meyer et al., 2021). Converging evidence has shown that neural oscillations at low and high-frequency ranges underlie language perception and production (Meyer et al., 2021, Poeppel and Assaneo, 2020, Ghitza et al., 2012, Abbasi, 2023), especially at theta-band frequencies (Ding, 2017). Moreover, the waveform of the speech signal is characterized by temporal regularity revealing an increase in power for frequencies between 2 and 8 Hz (Poeppel and Assaneo, 2020). Finally, a large body of work has shown that auditory cortices entrained by the speech envelope closely correspond to phonemic, syllables and prosodic information (Oganian and Chang, 2019) and that entrainment is modulated by intelligibility and comprehension (Riecke, 2018).

The potential role of neural oscillations during speech processing in ScZ is supported by a large body of work which has shown pronounced impairments in neural oscillations in both resting-state and task-related paradigms (Hirano and Uhlhaas, 2021), including during auditory processing (Grent-'t-Jong, 2023). Moreover, dysfunctional neural oscillations have involved speech production deficits at theta- and gamma-band frequencies (Ford, 2002, Ford, 2005), suggesting that altered neural oscillations may constitute a shared mechanism for both receptive and productive language impairments (Meyer et al., 2021).

At the cellular level, abnormalities in GABAergic interneurons as well as glutamatergic neurotransmission have been identified in auditory cortex (Addington, 2023) that mirror findings in prefrontal areas (Yung, 2005, Miller, 2002). Together, these data suggest a disruption in the balance between excitation and inhibition (E/I-balance) that could be relevant for language processing deficits in schizophrenia (Bell, 1994). Moreover, ScZ-patients are characterized by reduced gray matter (GM) volume across the language network, including both temporal and frontal cortices (Gross, 2013) and there is evidence for abnormal white matter connectivity within the dorsal pathway (Gross, 2013, Bedi, 2015).

In the current study, we investigated neural oscillations during speech- tracking in CHR-P participants and in patients with early-stage ScZ to examine the possibility that language networks in ScZ and during emerging psychosis are characterized by impaired speech entrainment. Given a proposed hierarchical phase-amplitude coupling between different frequencies that capture integration of lower-level phonological units into coherent higher-level percepts (Meyer, 2018), we added an attention manipulation. During one block participants focused on the discourse level (STORY condition) and in the other on the individual words (WORD condition), which would lead to a differential modulation of delta- and theta-band. Moreover, based on prior findings which have shown the central role of delta (1–4 Hz) and theta (4–7 Hz) band oscillations in speech comprehension (Gross, 2013) and the presence of speech anomalies in CHR-Ps and ScZ-patients (Perkins, 2015, Bedi, 2015, de Boer, 2023), we predicted that both CHR-P and ScZ-patients would be characterized by reduced low-frequency speech entrainment in auditory cortices.

## 2 Methods

### 2.1 Participants

We recruited a sample of 22 CHR-P, 23 early-stage ScZ patients and 44 healthy controls (HC). CHR-P participants were recruited from the general population through an online screening approach (see (McDonald, 2019) as part of the ongoing E-Detection Tool for Emerging Mental Disorders (ENTER) study funded by the Wellcome Trust. CHR-P status was confirmed through the Psyscan CHR-P interview (Addington, 2023) which includes the Comprehensive Assessment of At-Risk Mental State (CAARMS) instrument (Yung, 2005) and specifiers to additionally rate the Structured Interview for Psychosis-risk Syndromes (SIPS) (Miller, 2002) (Table 1).

**Table 1:** Demographics, Clinical Data, and Task performance

	**HC**	**CHR-P**	**SCZ**	**Main effect of Group**^a^	**Pairwise comparisons**
**Number of participants**	44	22	23		
**Age**, years (SD)	24.5 (4.7)	21.2 (3.7)	25.5 (4.9)	F(2,47.9) = 6.9, p = 0.002	CHR-P < HC, p = 0.010 SCZ > CHR-P, p = 0.005
**Sex**, male/female (%male)	20/24 (45.5)	1/21 (0.5)	17/6 (73.9)	X^2^(2) = 22.4, p < 0.001	HC > CHR-P, p < 0.001 HC < SCZ, p = 0.026 SCZ > CHR-P, p < 0.001
**Education**, years (SD)	18.3 (3.1)	16.1 (2.1)	15 (3.0)	F(2,48.1) = 8.1, p < 0.001	HC > CHR-P, p = 0.003 HC > SCZ, p = 0.006
**English Native language** (%)	20 (45.5)	16 (72.7)	14 (60.9)	No significant group differences	
**BACS**^b^, mean (SD)					
Verbal memory	−0.14 (0.9)	−0.29 (1.0)	−1.20 (1.0)	F(2, 43.2) = 9.2, p < 0.001	SCZ < HC, p < 0.001 SCZ < CHR-P, p = 0.011
Digit sequencing	−0.25 (1.6)	−0.78 (1.0)	−1.20 (1.6)	No significant group differences	
Token motor	0.00 (0.9)	−0.32 (1.4)	−0.78 (1.0)	F(2,41.0) = 5.2, p = 0.010	SCZ < HC, p = 0.007
Verbal fluency	0.01 (1.1)	0.34 (1.4)	−1.34 (1.1)	F(2,44.2) = 13.2, p < 0.001	SCZ < HC, p < 0.001 SCZ < CHR-P, p < 0.001
Symbol coding	−0.34 (1.1)	−0.72 (1.2)	−1.71 (0.8)	F(2, 48.2) = 18.1, p < 0.001	SCZ < HC, p < 0.001 SCZ < CHR-P, p = 0.006
Tower of London	0.32 (1.3)	−0.28 (1.3)	0.07 (1.8)	No significant group differences	
Composite score	−0.12 (1.0)	−0.58 (1.3)	−1.60 (1.3)	F(2, 42.5) = 11.6, p < 0.001	SCZ < HC, p < 0.001 SCZ < CHR-P. p = 0.025
**CAARMS**^c^, mean (SD)					
Unusual Thought Content	−	8.8 (8.7)	−		
Non-bizarre Ideas	−	12.2 (9.6)	−		
Perceptual Abnormalities	−	7.6 (7.2)	−		
Disorganized Speech	−	5.4 (1.1)	−		
Total severity score	−	35.9 (15.2)	−		
**SOFAS**, mean (SD)	−	65.6 (7.5)	53.1 (11.8)	F(1,37.5) = 18.2, p < 0.001	
**GF-role**, mean (SD)	8.0 (0.6)	7.6 (0.9)	5.9 (1.4)	F(2, 36.2) = 26.4, p < 0.001	SCZ < HC, p < 0.001 SCZ < CHR-P, p < 0.001
**GF-social**, mean (SD)	8.0 (0.9)	7.3 (0.7)	6.4 (1.1)	F(2,46.4) = 18.3, p < 0.001	SCZ < HC, p < 0.001 CHR-P < HC, p = 0.008 SCZ < CHR-P, p = 0.004
**PANSS**, mean (SD)					
Positive	−	−	11.5 (4.6)		
Negative	−	−	11.0 (3.5)		
Cognitive	−	−	12.2 (3.2)		
Excitement	−	−	4.6 (0.8)		
Depression	−	−	8.0 (2.5)		
Total score	−	−	47.3 (10.3)		
**Medication**^d^					
None	37	11	2		
Anti-depressives	0	8	5		
Mood stabilizers	0	0	0		
Anti-Psychotics	0	0	20		
Other	7	7	6		
**Task Performance**					
STORY accuracy (%, SD)	74.5 (18.9)	85.5 (11.0)	69.5 (20.6)	X^2^(2) = 7.7, p=0.021	CHR-P > SCZ: p-holm = 0.037
STORY confidence (%, SD)	82.4 (15.0)	90.6 (8.6)	78.7 (18.5)	X^2^(2) = 8.4, p=0.015	CHR-P > SCZ: p-holm = 0.019
WORD accuracy (%, SD)	65.6 (19.1)	67.4 (16.6)	53.9 (23.6)	No significant group differences	

Abbreviations: HC = healthy controls, CHR-P = Clinical High-Risk positive, SCZ = Schizophrenia patients, SD = standard deviation of the mean, GF = Global Functioning, SOFAS = Social Occupational Functioning Assessment Scale.

a All F-tests are Welch based; alpha=0.05, 2-sided, post-hoc Games-Howell or Holm (p-holm) corrected for Type I errors, X^2^ = Chi-square test.

b BACS scores for clinical groups were standardized to control group data from the Your Study (Uhlhaas, 2017), controlled for sex category

c Scores on subscales of the CAARMS represent global score times frequency score, severity the sum of those scores.

d If multiple medications were reported, they were scored separately in the different categories listed

ScZ-patients were recruited from the first-episode psychosis (FEP)-Service ESTEEM in Glasgow and were included if a) they reported the first treatment contact within a five year period and b) met criteria for ScZ on the Structured Clinical Interview for DSM-IV (Bell, 1994). Current psychotic symptoms were assessed with the Positive and Negative Symptom Scale (PANSS) (Kay et al., 1987) and symptoms were grouped into five factors according to the model of Lindenmayer et al. (Lindenmayer et al., 1995), including the factors “positive”, “negative”, “depression”, “excitement” and “cognitive”. Neurocognition was assessed with the Brief Assessment of Cognition in Schizophrenia (BACS) (Keefe, 1999).

The study was approved by the ethical committees of University of Glasgow and the National Health Services Research Ethical Committee Glasgow and Greater Clyde. Only right-handed participants were included. All participants provided written informed consent and were paid 6 pound Sterling per hour of participation.

## 3 Experimental Paradigm

Participants listened twice to a 5-minute recording of a Sherlock Holmes story (https://librivox.org/the-adventure-of-the-speckled-band-by-sir-arthur-conan-doyle/). The audio recording was bandpass filtered between 250–2500 Hz (steepness 10) using GoldWave audio software to factor in the band-limited frequency response of the long plastic tubes driven by MEG-compatable Etymotic system earplugs.

Participants listened to the recording while fixating the center of a translucent screen. In the first run, participants were instructed to listen to the story content (STORY condition). Subsequently, participants filled in a questionnaire containing 10 statements, rating on a scale from minus 100 (100 % false) to plus 100 (100 % true), thus measuring both accuracy of recall and confidence levels. In the WORD condition, participants were presented with the same recording, but were asked to press a button everytime they heard the word “THE”, while ignoring the story content.

## 4 Neuroimaging

MEG data were acquired on a TRIUX whole-head MEGIN system which comprises 102 sensors, each occupying one magnetometer and a set of two orthogonal gradiometers. A sampling rate of 1000  Hz was used and data were online filtered between 0.1 and 330 Hz. Prior to recordings, five head position coils (including the landmarks nasion and bilateral preauricular points) were placed and digitized together with the headshape using a Polhemus ^TM^ Fastrack system (Polhemus Inc., Vermont, USA). Locations of the coils were checked before and after each recording. Additionally, T1-weighted structural magnetic resonance images (MRIs) were obtained on a 3 Tesla scanner (Siemens, Tim Trio System) using a 3D Magnetization Prepared Rapid Gradient Echo sequence. The parameters were: 1 × 1 × 1 mm resolution, 192 volumes, TR = 2.250 ms, TE = 2.6 ms, FA = 9°.

### 4.1 Data analysis

To estimate individual speech-tracking ability, a Gaussian-Copula Mutual Information (GCMI) analysis (Ince, 2017), which computes statistical dependence between the MEG and the speech envelope, was used. We used GCMI, rather than typical correlation analyses, due to several advantages of this method, because: 1) it captures non-normally distributed dependencies (e.g., asymmetric or tail dependencies), using rank-based data, 2) it is computationally efficient, and 3) it does not require large amounts of data (Ince, 2017). Preprocessing included the computation of the amplitude envelope of the auditory signals using the Matlab Chimera toolbox and standard routines (Gross, 2013). First, audio data were splitted into nine frequency bands in the range 100–10.000 Hz, equidistant on the cochlear map (Smith et al., 2002). Data were bandpass filtered using a 4th order Butterworth filter (two directions), and amplitude envelopes were computed for each band as absolute values of the Hilbert transform. These were subsequently averaged across bands to obtain a wide-band amplitude envelope data and downsampled to 250 Hz for further GCMI analysis.

In the MEG data, sensors with excessive artefacts were identified, which were then corrected using MNE-Phyton routines prior to submitting the data to temporal Signal-Space Separation (tSSS, Maxfilter) method (Taulu and Simola, 2006) to suppress contribution of magnetic signals from external sources. Only magnetometers were used for further analyses.

In the next step, one continuous trial of the same length of the soundtrack was created for each sensor, while correcting a slight mismatch in the timing of the acquisition and stimulus presentation hardware by adjusting the sampling rate of the MEG data to 249.8671 Hz. Data were subsequently demeaned and corrected for eyeblinks, eye movement, and heart-beat contamination using fastICA decomposition and removal routines from the Matlab Toolbox Fieldtrip (version 20221223: (Oostenveld, 2011).

Data were further processed in source space using a virtual channel approach. First, individual T1-weighted MRIs were co-registered with MEG data using three anatomical landmarks (nasion and bilateral pre-auricular points), followed by an automatic more fine-grained co-registration procedure with the ICP algorithm (Besl and McKay, 1992). The co-registered MRI data were segmented into white/grey matter (WM/GM) and CSF before applying a single-shell volume conductor model to compute the individual head model. A source model grid was based on a normalized individual MRI in a 5 mm template MRI (Montreal Neurological Institute, MNI).

**Fig. 1:**
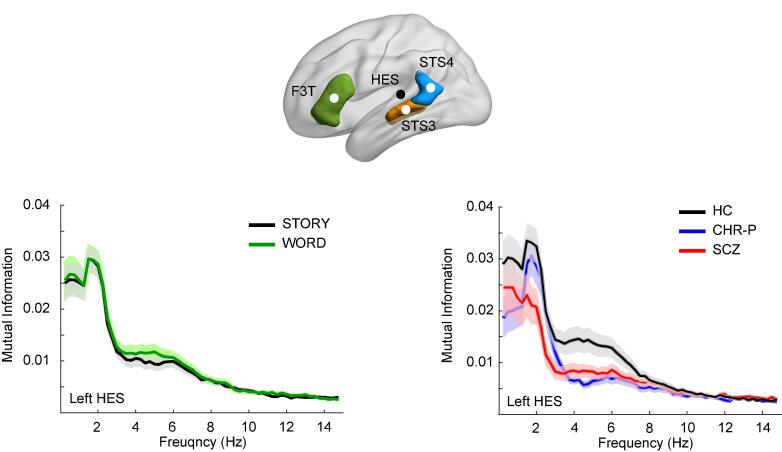
MEG results. Top figure panel shows the regions of interest, projected on the left side of a brain surface generated with Brain Netviewer software (Xia et al., 2013). Heschl’s gyrus (HES) location, black dot, was identified manually from anatomical T1 scans, whereas the central nodes in the three AICHA atlas regions (F3T, STS3, STS4) was based on locations published (Labache, 2019). Lower left panel shows the mutual information left Heschl’s gyrus (LHES) spectra for the main effect of CONDITION, averaged over GROUPs, with the STORY condition shown in black and the WORD condition in green. The lower right panel shows the main effect of GROUP, averaged over CONDITIONs in LHES, separately per group with HCs in black, CHR-Ps in blue, and early SCZ patients in red. Error bars depict standard error of the mean. Abbreviations: HC = healthy control; CHR-P = Clinical High-Risk positive; SCZ = schizophrenia patient.

Eight regions-of-interest (ROIs) were selected for virtual-channel single-trial time series reconstruction, using LCMV beamformers (Van Veen, 1997) with a regularisation parameter of 20 % to attenuate leakage from nearby sources. ROIs included bilateral Heschl’s gyrus (LHES, RHES), and central nodes in 3 bilateral AICHA atlas regions (see Fig. 1A) identified in the SENSAAS atlas SENT_CORE network as central hubs in processing of auditory speech data (Labache, 2019). These included F3T (Frontal_Inf_Tri_1; Broca’s areas; LF3T, RF3T), STS3 (Superior_Temporal_3; anterior part of Wernicke’s areas; LSTS3, RSTS3) and STS4 (Superior_Temporal_4; posterior part of Wernicke's areas; LSTS4, RSTS4).

MI analysis was applied to the Fourier transformed soundtrack envelope and virtual-channel MEG data for frequencies between 0.25 and 50 Hz, using 0.25 Hz resolution and Slepian multitapers with a smoothing factor of 4 Hz. Prior to these analyses, the MEG data were shifted by 100 ms as in previous studies (Park, 2018) to compensate for delays between the brain response and auditory input.

Finally, MEG responses of CHR-Ps were correlated with symptom scores, using a linear regression with stepwise method to exclude non-contributing factors, including MEG-data as dependent variable and covariates. These included CAARMS subscales for Unusual Thought Content (UTC), Non-Bizare Ideas (NBI), Perceptual Abnormalities (PA) and Disorganized Speech (DS) as well as the Social Occupational Functioning Assessment Scores (SOFAS).

## 5 Statistics

Statistical analyses were performed in JASP software (JASP Team (2024), JASP Version 017.2.1; https://jasp-stats.org/). For demographic, clinical and neuropsychological variables, one-way Welch ANOVAs were used to test for group differences. BACS data were first Z-score standardized to HCs from an existing sample while controlling for sex. Alpha levels were 0.05 and 2-sided.

All MEG and behavioral data were first tested for violations of normal distribution, using Shapiro-Wilk tests. Assumptions of normal distribution were violated for all these data. Therefore, we used non-parametric Kruskal-Wallis and post-hoc Dunn’s tests for behavioural analysis of GROUP differences in accuracy and confidence levels. MEG data were first log10 transformed and then submitted to repeated-measure ANCOVA’s in a GROUP x CONDITION design, including the between-subject factor GROUP (HC, CHR-P, ScZ), the within-subject factor CONDITION (STORY, WORD), as well as the covariate SEX (female, male). Post-hoc comparisons were Holm corrected (p-holm) for multiple comparisons. For the 8 ROI virtual-channel data, only delta (0.25–2.00 Hz) and theta (4.50–6.50 Hz) band activity was tested, averaged over frequencies within each band.

## 6 Results

### 6.1 Demographic and neuropsychological data

Groups differed in age (p < 0.005), years of education (p < 0.001), percentage of male participants (p < 0.001), but not in percentage of native speakers. CHR-Ps were younger than both HCs and ScZ-patients while HCs had more years of education.

BACS composite scores where lower in ScZ-patients compared to both HCs (p < 0.001) and CHR-Ps (p = 0.025). Impairments included significantly lower scores on verbal memory (p < 0.001), motor speed (p = 0.007), verbal fluency (p < 0.001) and symbol coding task (p < 0.001). SOFAS scores were significantly lower in the ScZ-groups compared to CHR-Ps. ScZ-patients were also characterized by lower Global Function Role (GF-role) and social (GF-social) scores, compared to both HCs and CHR-Ps.

### 6.2 Task performance

CHR-Ps had higher scores than ScZ-patients on STORY recall (p = 0.029), but both clinical groups were not different from HCs. The groups did also not differ in STORY recall nor in word-detection accuracy in the WORD condition.

### 6.3 MEG analyses

Repeated-measures ANCOVA’s revealed significant findings for theta-band responses in LHES, including a main effect of CONDITION (F(1,85) = 5.5, p = 0.022, partial ƞ^2^ = 0.060), a main effect of GROUP (F(2,85) = 3.5, p = 0.033, partial ƞ^2^ = 0.077), and a trend-level GROUP x CONDITION interaction effect (F(2,85) = 3.0, p = 0.054, partial ƞ^2^ = 0.067). SEX and CONDITION x SEX were not significant. The main effect of CONDITION, averaged over GROUP, resulted from THETA responses being larger in the WORD than STORY condition. Post-hoc testing for the main effect of GROUP effect, averaged over CONDITION and controlled for SEX, showed that the LHES-theta responses (Fig. 1) were in CHR-Ps were significantly lower than those from the HCs (t = -2.5, p-holm = 0.045, Cohen’s d = 0.65), whereas the responses in early ScZ patients were normal (p = 0.303). No such differences were found in any other ROI, nor in the delta frequency range.

### 6.4 MEG analyses: Correlations with symptom scores

**Fig. 2:**
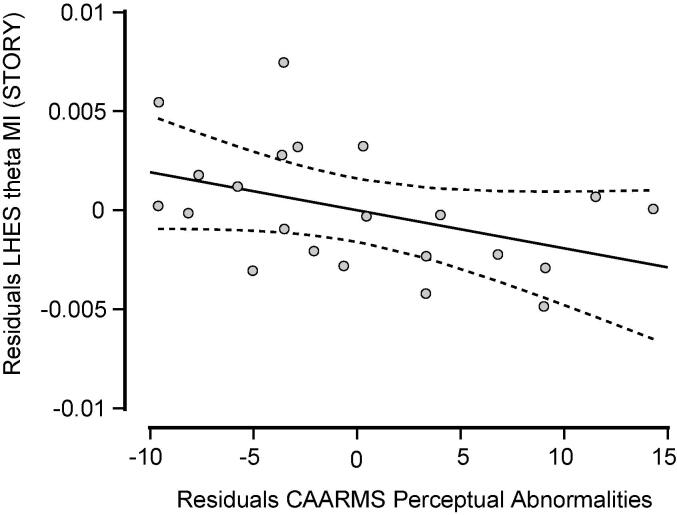
Partial correlation plot, showing the correlation between LHES theta-band responses in CHR-Ps and their CAARMS Perceptual Abnormality scores. Abbreviations: CAARMS = Comprehensive Assessment of At-Risk Mental State; LHES = left Heschl’s gyrus; CHR-Ps = Clinical High-Risk positive individuals.

A linear regression analyses was performed between LHES theta-band activity and symptoms scores in the CHR-P group. A significant model (*F*(1,21) = 6.7, p = 0.018, adj. R-square = 0.212) with the contribution of the CAARMS “Perceptual Abnormalities” scale was found (t = -2.6, p = 0.018, standardized beta coefficient = -0.500) (Fig. 2).

## 7 Discussion

Alterations in language are a core aspect of ScZ which may have the potential to provide insights into the pathophysiology of the disorder (Brown and Kuperberg, 2015, Chang, 2022) as well as biomarkers for early detection and diagnosis (Corcoran and Cecchi, 2020). In the current study, we investigated neural oscillations during continuous speech to examine the hypothesis that oscillatory entrainment is impaired in early-stage ScZ and CHR-P participants. Our results show that only CHR-Ps were characterized by impaired tracking of the speech envelope at theta-band frequencies, suggesting that decreased entrainment of auditory cortex may underlie dysfunctional language processing in emerging psychosis.

Consistent with previous findings (Gross, 2013), we observed low-frequency entrainment to continuous speech, in particular in LHES, suggesting a functional role of primary auditory cortex in the processing and transforming simple to complex acoustic features (Khalighinejad, 2021). Importantly, entrainment at 4.5–6.5 Hz was impaired only in CHR-Ps. Previous studies during normal brain functioning have linked theta-band entrainment to phonetic processing as well as tracking of syllable rate (Poeppel and Assaneo, 2020) while entrainment at delta-frequencies has been linked to the encoding of more complex features, such as syntactic structures (Keitel et al., 2018).

Deficits were most prominent in the CHR-P group when participants were required to attend to the story content as opposed to the world level. Previous studies have shown that ScZ-patients are chacterized by pronounced impairments during discourse comphrehension vs. lexical processing (Swaab, 2013) which is consistent with the view that deficits in language processing in ScZ may be due to impairments in generative processes between higher-level and low-level perceptual representations (Brown and Kuperberg, 2015). Interestingly, elevated perceptual abnormalities in CHR-P participants correlated with impaired theta-band power in LHES, suggesting that attenuated psychotic symptoms and decreased entrainment of auditory cortex may stem of overlapping circuit deficits.

Previous studies (Ford, 2002, Ford, 2005) demonstrated impaired low-and high-frequency coherence between frontal and temporal areas during speech production in ScZ-patients. Moreover, there is evidence for the contribution of high-frequency oscillations to low-level speech processing deficits in ScZ (Hirano, 2008). Together, these findings highlight the possibility that low- and high-frequency neural oscillations could reflect a parsimonious mechanism to account for both productive and receptive speech impairments in ScZ and possibly in emerging psychosis.

Future studies should extend the current analytic framework by investigating relationships between delta- and theta-band tracking in response to syllables and phonemes of the speech envelope [62]. Moreover, there is evidence for a top-down contribution during speech tracking from inferior frontal areas and motor areas to auditory cortex during normal brain functioning [63] which could potentially be relevant for accounting for impairments in primary auditory cortex observed in the current study. In addition, recent studies using natural language processing (NLP) approaches have indicated reduced discourse coherence, syntactic complexity and language connectedness in CHR-Ps which predict transition to psychosis (Corcoran, 2020). Accordingly, studies should investigate the possibility of using EEG/MEG-data during language processing to predict clinincal outcomes in CHR-P and possibly also first-episode ScZ.

## 8 Summary

The current study provides novel evidence for the hypothesis that auditory cortex in CHR-Ps, but not in early-stage ScZ, is characterized by impaired theta-band entrainment, suggesting that neural oscillations could account for dysfunctional language abnormalities. The presence of reduced oscillatory entrainment in CHR-Ps furthermore highlights the possibility that neurophysiological markers of speech processing may have potential as biomarkers for early detection and diagnosis. Future studies should therefore combine oscillatory entrainment with more detailed analyses of linguistic and semantic markers to reveal the neurophysiological origins of dysfunctional language processing in ScZ and their potential utility for early detection and diagnosis.

## 9 Financial Disclosure

Dr. Uhlhaas has received research support from Lilly and Lundbeck outside the submitted work. Drs. Fusar-Poli, Grent-'t-Jong, Gross, Gumley, Muckli, Dheerendra and Krishnadas report no conflict of interest.

## CRediT authorship contribution statement

**Tineke Grent-’t-Jong:** Writing – review & editing, Writing – original draft, Formal analysis, Data curation, Conceptualization. **Pradeep Dheerendra:** Writing – review & editing, Formal analysis, Data curation, Conceptualization. **Paolo Fusar-Poli:** Writing – review & editing, Funding acquisition. **Joachim Gross:** Writing – review & editing, Software, Methodology, Conceptualization. **Andrew I. Gumley:** Writing – review & editing. **Rajeev Krishnadas:** Writing – review & editing, Funding acquisition. **Lars F. Muckli:** Writing – review & editing, Funding acquisition. **Peter J. Uhlhaas:** Writing – review & editing, Writing – original draft, Supervision, Resources, Project administration, Funding acquisition, Conceptualization.

## Data Availability

Data will be made available on request.
